# Lasso-Enhanced Logistic Regression for Early Prediction of Pulmonary Infection in Critically Ill Post-Abdominal Surgery Patients

**DOI:** 10.3390/medicina62040788

**Published:** 2026-04-20

**Authors:** Bin Wang, Jie Zhao, Fengxue Zhu

**Affiliations:** 1 Department of Critical Care Medicine, Peking University People’s Hospital, Beijing 100044, China; 2 Harvard Medical School, Massachusetts General Hospital, Boston, MA 02114, USA; 3 Trauma Medicine Center, Peking University People’s Hospital, Beijing 100044, China

**Keywords:** ICU, abdominal surgery, pulmonary infection, predictors, risk stratification model

## Abstract

*Background and Objectives:* To identify predictors of pulmonary infection in critically ill patients after abdominal surgery and to develop an early postoperative risk stratification model. *Materials and Methods:* Medical records of ICU patients after abdominal surgery (January 2016–June 2024) with Acute Physiology and Chronic Health Evaluation II (APACHE II) scores ≥10 were retrospectively analyzed. Patients were categorized according to the presence or absence of pulmonary infection. Candidate variables were screened using LASSO regression, followed by multivariate logistic regression to identify independent predictors. A nomogram-based prediction model was constructed and internally validated. *Results*: Among 4852 patients, 390 (8.0%) developed pulmonary infections. Overall, 8 independent predictors were identified: Male sex (vs. female) (OR 1.509, 95% CI: 1.091–2.087, *p* = 0.013), chronic obstructive pulmonary disease (OR 4.139, 95% CI: 2.872–5.966, *p* < 0.001), atrial fibrillation (OR 2.320, 95% CI: 1.366–3.939, *p* = 0.002), hypertension (OR 1.869, 95% CI: 1.372–2.539, *p* < 0.001), chronic renal insufficiency (OR 2.412, 95% CI: 1.143–5.091, *p* = 0.021), preoperative total bilirubin (OR 1.003, 95% CI: 1.001–1.004, *p* = 0.002), rectal surgery (OR 0.354, 95% CI: 0.151–0.830, *p* = 0.017), and invasive mechanical ventilation duration > 6 h (OR 2.206, 95% CI: 1.628–2.990, *p* < 0.001). The nomogram demonstrated good discrimination (AUC: 0.734 95% CI: 0.698–0.770) and calibration. *Conclusions*: This study identified 8 independent predictors of pulmonary infection and developed an internally validated early postoperative risk stratification model with satisfactory performance. The model may assist clinicians in identifying high-risk patients and guiding timely preventive strategies in ICU practice.

## 1. Background

The abdominal cavity contains multiple vital organs, leading to a high incidence of abdominal diseases that often require surgical treatment. Despite advances in perioperative care, postoperative complications remain common, particularly among critically ill patients. Pulmonary infections are a major concern, as postoperative pain, reduced mobility, and weakened respiratory function impair airway clearance and markedly increase susceptibility to infection [1]. These risks are further amplified in intensive care unit (ICU) patients, who typically have limited physiological reserve and face substantial clinical challenges [1].

Pulmonary infection is a common and serious postoperative complication among critically ill patients following abdominal surgery [2]. Pulmonary infection can range from mild inflammatory responses to severe respiratory failure and multi-organ dysfunction, with reported mortality rates of 9–50% and substantially increased healthcare costs [3,4,5].

Abdominal surgery encompasses a broad spectrum of procedures involving upper and lower abdominal organs [6]. The heterogeneity of surgical techniques and patient conditions complicates early identification of individuals at high risk for postoperative pulmonary infection. Although several predictive models have been proposed, few have specifically targeted critically ill patients after abdominal surgery [7,8].

## 2. Methods

### 2.1. Study Population

**Figure 1:**
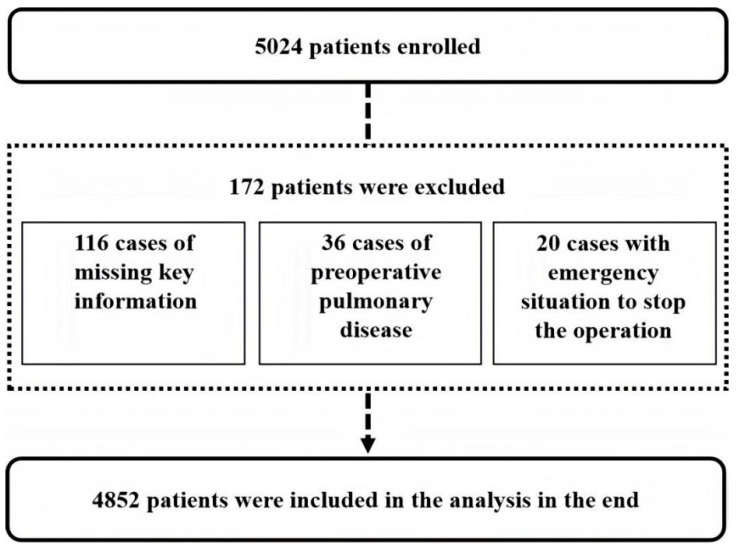
Study flowchart.

This retrospective study included critically ill patients admitted to the ICU after abdominal surgery at Peking University People’s Hospital between 1 January 2016 and 30 June 2024. A total of 4852 patients met the inclusion criteria (Figure 1). Clinical data were accessed on 15 May 2025. Investigators had access to identifiable information (e.g., medical record numbers) during data extraction within the hospital information system. All data were fully de-identified before statistical analysis.

In our study, pulmonary infection was defined as pneumonia occurring more than 48 h after surgery during hospitalization [9]. Pulmonary infection was diagnosed when new or progressive pulmonary infiltrates were present on chest imaging and the patient met at least two of the following clinical criteria:

1. Fever > 38.0 °C or hypothermia < 36.0 °C
2. Leukocytosis > 12 × 10^9^/L or leukopenia < 4 × 10^9^/L
3. New onset of purulent sputum, increased sputum production, or worsening cough
4. Worsening oxygenation, defined as increased FiO_2_ or PEEP requirement
5. Positive respiratory culture (sputum, tracheal aspirate, or BAL), if available

To avoid misclassification, non-infectious causes (atelectasis, pulmonary edema, aspiration without infection, pulmonary embolism) were excluded based on clinical judgment. This definition was adapted from the European Perioperative Clinical Outcome (EPCO) criteria and CDC/NHSN guidelines [2,9,10,11].

Inclusion criteria were:

1. Age ≥ 18 years;
2. Direct ICU admission after abdominal surgery;
3. Acute Physiology and Chronic Health Evaluation II (APACHE II) score ≥ 10 [12].

Exclusion criteria were:

1. Missing or incomplete electronic medical records;
2. Presence of pulmonary infection prior to surgery;
3. Surgeries aborted or discontinued due to intraoperative emergencies such as respiratory or cardiac arrest.

The study was conducted in accordance with the principles of the Declaration of Helsinki and was approved by the Ethics Committee of Peking University People’s Hospital (Approval No. 2025PHB236-001). Informed consent was waived due to the retrospective design of the study.

### 2.2. Data Collection

Patient data were systematically collected and included the following categories:

Demographics: gender, age, and body mass index (BMI). Comorbidities and medical history: chronic obstructive pulmonary disease (COPD), asthma, atrial fibrillation, coronary heart disease, diabetes mellitus, hypertension, cerebral infarction, chronic renal insufficiency, hepatitis B virus (HBV) infection, fatty liver disease, history of chemotherapy, and smoking status. Laboratory examinations: preoperative leukocyte count, hemoglobin, platelet count, albumin, creatinine, and total bilirubin; postoperative creatine kinase MB (CK-MB), B-type natriuretic peptide (BNP), prothrombin time (PT), and activated partial thromboplastin time (APTT). Surgical and anesthesia-related indicators: type of surgery (pancreatic, biliary, liver, splenic, jejunal, ileal, colonic, rectal, renal, ureteral, bladder, uterine, ovarian), laparoscopic versus open approach, night-time surgery, intraperitoneal chemotherapy, intraoperative thermoperfusion therapy, microwave ablation, operation duration, total intraoperative fluid intake and output, intraoperative blood loss, transfusion requirements, vasoactive drug infusion, use of nerve block, analgesic pump, nasogastric tube placement, American Society of Anesthesiologists (ASA) grade ≥ III, and Invasive mechanical ventilation duration > 6 h.

Due to the retrospective design, laboratory indicators were not consistently measured at both preoperative and postoperative time points. Therefore, variables were selected based on clinical relevance and data availability. Postoperative laboratory samples were obtained within 2 h after ICU admission to capture the patient’s immediate postoperative physiological status. These variables were incorporated to support early postoperative risk stratification.

### 2.3. Statistical Approach

Data analysis was performed using R software (version 4.0.3). Continuous variables were expressed as mean ± standard deviation (SD) or median with interquartile range (IQR), depending on data distribution. Data normality was assessed using the Shapiro–Wilk test and by visual inspection of Q–Q plots. Categorical variables were summarized as frequencies and percentages.

Lasso regression was applied to reduce dimensionality and identify variables with significant associations. Subsequently, multivariate logistic regression was conducted to determine independent predictors for pulmonary infection. Based on these factors, a nomogram was constructed to visualize the predictive model.

Model performance was evaluated using the area under the receiver operating characteristic (ROC) curve. Calibration curves were generated to assess agreement between predicted and observed outcomes, while decision curve analysis (DCA) was performed to evaluate clinical utility. A two-sided *p*-value < 0.05 was considered statistically significant.

Missing data were handled using complete-case analysis. Given the large sample size, this approach was considered acceptable. Sensitivity analyses comparing patients with and without missing data showed no substantial differences in baseline characteristics or outcomes.

## 3. Result

A total of 4852 patients met the inclusion criteria, of whom 390 (8.0%) developed pulmonary infections. Compared with the non-infection group, patients with pulmonary infection demonstrated significantly higher in-hospital mortality (15.1% vs. 2.9%), longer ICU stays (median [IQR]: 2 [2–5] days vs. 2 [2–3] days), extended overall hospitalization (26 [17–40] days vs. 18 [14–24] days), and greater total hospitalization costs (198 [114–328] thousand yuan vs. 114 [81–165] thousand yuan; all *p* < 0.001) (Table 1). These findings highlight the considerable clinical and economic burden associated with pulmonary infections in critically ill patients following abdominal surgery.

### 3.1. Comparison Between Infection and Non-Infection Groups

Between-group comparisons using χ^2^ tests, *t*-tests, or Mann–Whitney U tests identified multiple variables with *p* < 0.10, which were subsequently considered for LASSO regression. These included demographic factors (gender, age), comorbidities (chronic obstructive pulmonary disease [COPD], atrial fibrillation, diabetes mellitus, hypertension, cerebral infarction, chronic renal insufficiency, smoking), laboratory parameters (preoperative leukocyte count, hemoglobin, platelet count, albumin, total bilirubin; postoperative CK-MB, BNP, and APTT), and surgical/anesthesia-related indicators (pancreatic surgery, rectal surgery, kidney surgery, uterine surgery, ovarian surgery, laparoscopic surgery, night-time surgery, intraoperative thermoperfusion therapy, total intraoperative output, intraoperative blood loss, intraoperative transfusion, intraoperative vasoactive drug infusion, nasogastric tube placement, ASA grade ≥ III, and Invasive mechanical ventilation duration > 6 h) (Table 2).

### 3.2. Lasso Regression

**Figure 2:**
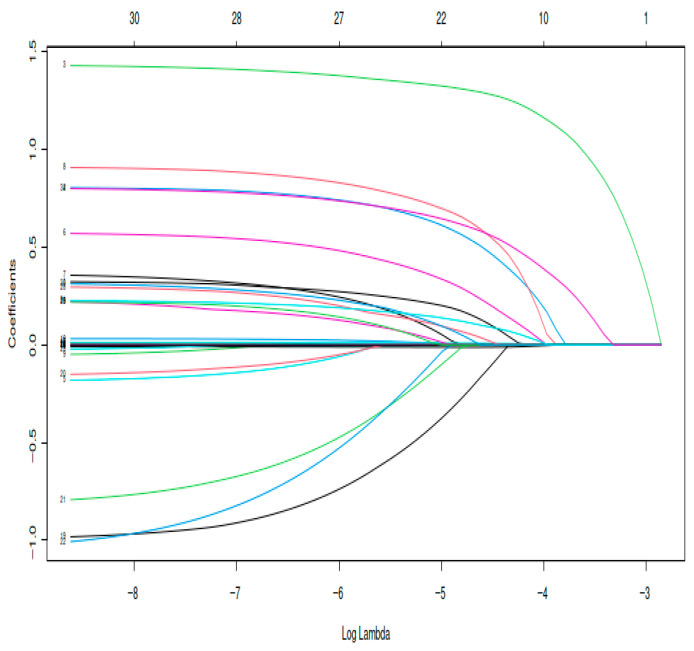
Coefficient path plot of the LASSO regression model. Note: Regularization paths of candidate predictors across a sequence of penalty parameters. Ten variables with non-zero coefficients were retained at the optimal tuning parameter (λ = 0.0101), selected via cross-validation. Different colors represent individual predictor variables, with each line corresponding to the coefficient trajectory of one variable. Colors are used solely to distinguish variables and do not carry specific meaning.

**Figure 3:**
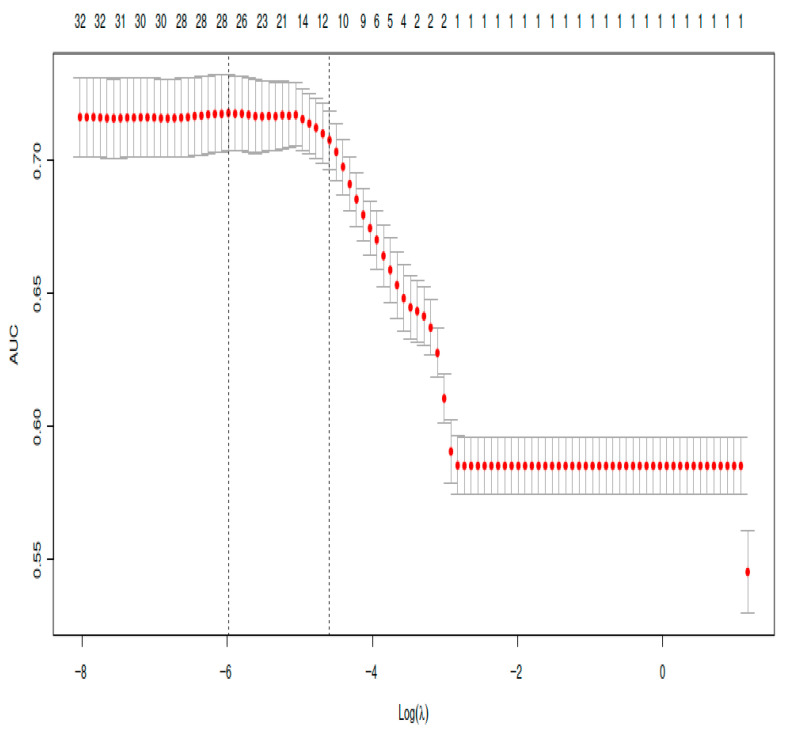
Cross-validation curve for selecting the optimal penalty parameter (λ) in the LASSO model. Note: Mean AUC values with standard errors across a sequence of λ values. The optimal λ (0.0101) was selected using the one-standard-error rule.

Lasso regression was applied to reduce the dimensionality of the dataset and identify variables associated with pulmonary infection. Ten variables with non-zero coefficients were retained (Figure 2). The optimal penalty parameter (λ = 0.0101, λ.1se) was selected through cross-validation (Figure 3). The variables identified included gender, chronic obstructive pulmonary disease (COPD), atrial fibrillation, hypertension, chronic renal insufficiency, preoperative hemoglobin, preoperative platelet, preoperative albumin, preoperative total bilirubin, postoperative B-type natriuretic peptide (BNP), rectal surgery, postoperative activated partial thromboplastin time (APTT), American Society of Anesthesiologists (ASA) grade ≥ III, transfusion, nasogastric tube and Invasive mechanical ventilation duration > 6 h (Figure 2).

### 3.3. Multivariate Logistic Regression

Multivariate logistic regression identified 8 independent predictors for pulmonary infection: Male sex (vs. female) (OR 1.509, 95% CI: 1.091–2.087, *p* = 0.013), chronic obstructive pulmonary disease (OR 4.139, 95% CI: 2.872–5.966, *p* < 0.001), atrial fibrillation (OR 2.320, 95% CI: 1.366–3.939, *p* = 0.002), hypertension (OR 1.869, 95% CI: 1.372–2.539, *p* < 0.001), chronic renal insufficiency (OR 2.412, 95% CI: 1.143–5.091, *p* = 0.021), preoperative total bilirubin (OR 1.003, 95% CI: 1.001–1.004, *p* = 0.002), rectal surgery (OR 0.354, 95% CI: 0.151–0.830, *p* = 0.017), and invasive mechanical ventilation duration > 6 h (OR 2.206, 95% CI: 1.628–2.990, *p* < 0.001) (Table 3).

### 3.4. Nomogram and Model Validation

**Figure 4:**
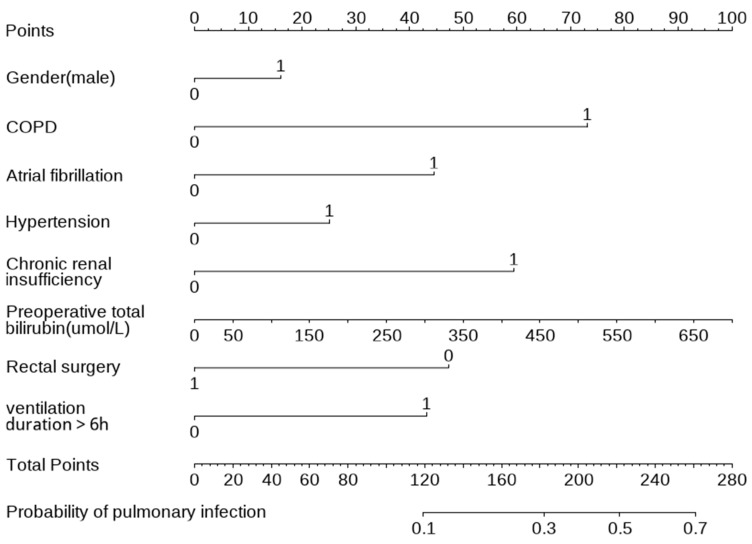
Nomogram predictive model. COPD: chronic obstructive pulmonary disease.

**Figure 5:**
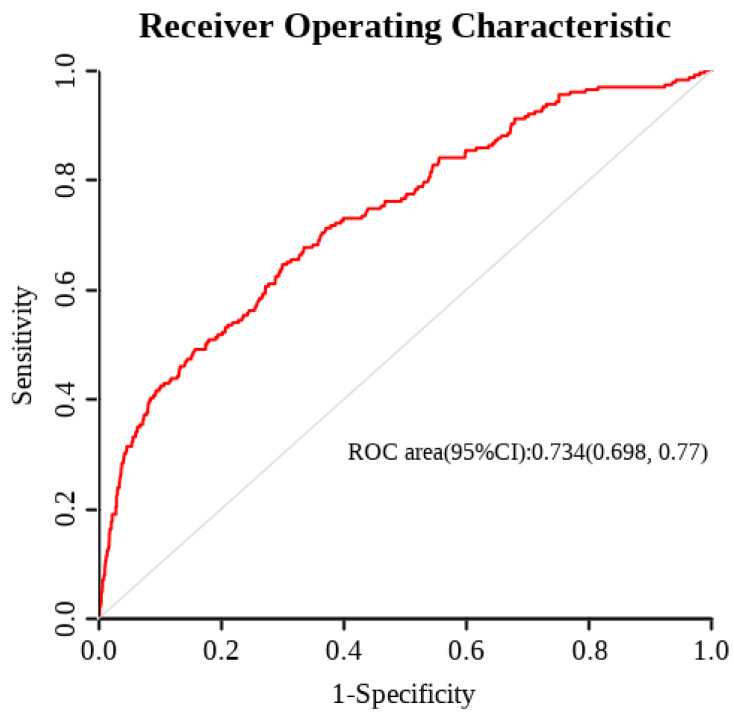
ROC curve. Note: The model demonstrated good discrimination, with an area under the ROC curve (AUC) of 0.734 (95% CI: 0.698–0.770).

A nomogram was developed based on the 8 identified risk factors to predict the probability of pulmonary infection (Figure 4). The model demonstrated good discriminatory ability, with an area under the receiver operating characteristic (ROC) curve (AUC) of 0.734 (95% CI: 0.698–0.770) (Figure 5).

**Figure 6:**
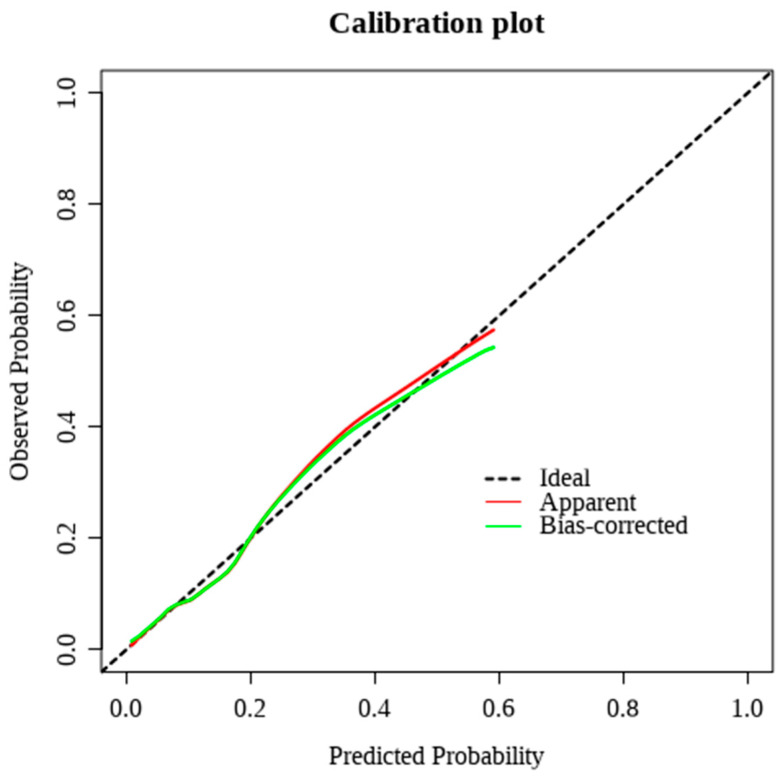
Calibration curve. The apparent and bias-corrected curves demonstrate good concordance between predicted and observed risks. The bias-corrected curve was obtained via 500 bootstrap resamples to adjust for optimism. Calibration slope and intercept were derived from the bootstrap-corrected model.

Calibration curves demonstrated good agreement between predicted and observed outcomes (Figure 6). After internal validation with 500 bootstrap resamples, the optimism-corrected calibration intercept was 0.05, indicating minimal systematic bias. The calibration slope was 0.79, reflecting mild shrinkage of predicted probabilities and overall acceptable calibration performance.

**Figure 7:**
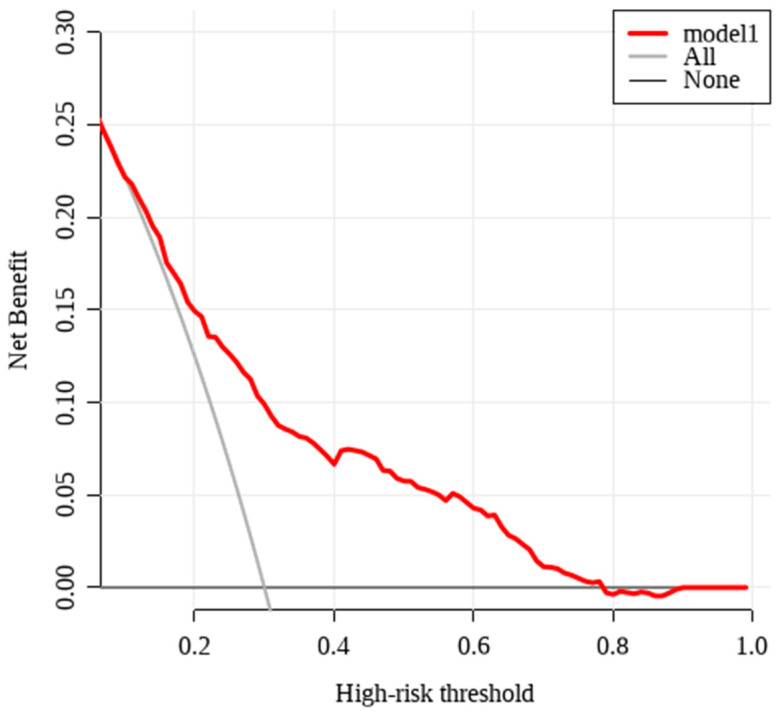
Decision Curve Analysis. The decision curve shows that the predictive model provides a higher net benefit than the “Treat All” and “Treat None” strategies across a wide range of threshold probabilities, supporting its clinical utility.

Decision curve analysis (DCA) further confirmed the clinical utility of the model, showing superior net benefit compared with both the “Treat All” and “Treat None” strategies (Figure 7).

Note: The nomogram integrates 8 independent predictors—Gender (male), COPD, atrial fibrillation, hypertension, chronic renal insufficiency, preoperative total bilirubin, rectal surgery, and mechanical ventilation > 6 h—to estimate the probability of pulmonary infection. For use, locate each patient’s value on the corresponding variable axis, draw a vertical line upward to obtain the point score, sum all points to derive the total score, and project the total score downward to determine the predicted risk. COPD: chronic obstructive pulmonary disease.

## 4. Discussion

Pulmonary infection is a frequent and serious postoperative complication, particularly among critically ill patients following abdominal surgery, and is associated with increased mortality, prolonged hospitalization, and elevated healthcare costs [6]. Previous studies have reported an incidence of 2.8% after open abdominal surgery [7]. In contrast, the incidence observed in our study was higher, reaching 8%, which may be attributed to the severity of the patient population and is comparable to the 9.6% incidence reported in our earlier work [13].

Moreover, in a study including both chest and abdominal surgeries, the incidence of pulmonary infection was 17.5% [7]. Although the definition of infection in that study was broader, the findings suggest that procedures closer to the lungs—such as thoracic and upper abdominal surgeries—may exert a greater impact on postoperative pulmonary complications. Consistent with previous reports, patients who developed pulmonary infection in our cohort demonstrated significantly worse prognostic indicators, including higher mortality, longer ICU and hospital stays, and greater hospitalization costs, underscoring the substantial clinical and economic burden of this complication.

The study identified 8 relevant factors for postoperative pulmonary infection: gender, COPD, atrial fibrillation, hypertension, chronic renal insufficiency, elevated preoperative total bilirubin, rectal surgery, and invasive mechanical ventilation > 6 h.

In our study, male sex (vs. female) was independently associated with an increased risk of postoperative pulmonary infection. Growing evidence suggests that male patients have an intrinsically higher susceptibility to pulmonary infection, which aligns with the findings of our postoperative prediction model. Population-based studies have shown that men experience more severe community-acquired pneumonia and worse clinical outcomes compared with women, indicating sex-related differences in host defense mechanisms [14]. Broader epidemiological analyses further demonstrate that males have a higher incidence and severity of lower respiratory tract infections, potentially related to hormonal modulation of immune responses and differences in mucosal immunity [15]. Surgical data reinforce this pattern. A Japanese multicenter study of 3979 colorectal cancer patients identified male sex as an independent predictor of postoperative pulmonary complications (OR 2.165) [16], consistent with findings from major oral cancer surgery showing similar sex-related vulnerability [17]. These converging data support a biologically plausible and clinically relevant association between male sex and postoperative pulmonary infection. Recognizing this risk factor may enhance early stratification and guide targeted perioperative prevention strategies.

COPD emerged as a strong risk factor, consistent with previous studies emphasizing its inflammatory nature and its role in airway obstruction and impaired immune responses [7]. As a chronic inflammatory condition characterized by persistent airway obstruction, its prevalence continues to rise worldwide [18,19]. Patients with COPD typically experience progressive decline in lung function, structural damage to the airway mucosa, and reduced immune competence, all of which increase their susceptibility to pulmonary infections.

Atrial fibrillation is a common arrhythmia, mainly characterized by irregular and rapid heart rate. In severe cases, it can lead to heart failure or thromboembolism, threatening the life of patients [20,21]. Pulmonary infection is one of the predisposing factors of atrial fibrillation, and the decrease in heart function can increase the probability of pulmonary infection in patients with atrial fibrillation [22]. Studies have found that inflammation and immune function can mediate the occurrence, development and deterioration of atrial fibrillation, and some inflammatory response pathways leading to atrial fibrillation may also lead to immune dysfunction in patients and make them more susceptible to infection [23,24].

In this study, hypertension was identified as an independent risk factor for pulmonary infection. Although there is generally no direct causal relationship between hypertension and pulmonary infection, patients with hypertension often exhibit impaired cardiac function, vascular integrity, and microcirculatory regulation compared with the general population. These physiological alterations may predispose hypertensive patients to a higher risk of postoperative pulmonary complications [25].

Patients with chronic renal insufficiency are prone to complications such as fluid overload and pulmonary edema, which increase their susceptibility to lung infection. In addition, impaired immune function in these patients not only predisposes them to infection but also contributes to poorer outcomes, including deterioration of renal function, adverse drug reactions, and higher case fatality rates compared with the general population [26,27].

The impact of preoperative bilirubin elevation on pulmonary infection has been rarely reported in previous studies. Nevertheless, extensive evidence indicates that patients with liver dysfunction are more susceptible to infectious events and edema due to impaired immunity, and pulmonary edema has been reported in more than 30% of patients with liver failure [28]. As bilirubin is a representative marker of liver function, its elevation emerged as an independent risk factor for pulmonary infection in this study. Furthermore, most patients with hyperbilirubinemia suffer from hepatobiliary diseases, and their surgeries are typically hepatobiliary procedures. These operations, located near the diaphragm and technically complex, are more likely to result in postoperative pulmonary complications [25].

Our finding that rectal surgery was associated with a lower risk of postoperative pulmonary infection is clinically plausible. Surgical procedures involving the upper abdomen are known to impair diaphragmatic excursion, increase postoperative pain, and predispose patients to atelectasis and pneumonia, whereas pelvic and lower abdominal operations exert less impact on respiratory mechanics [29]. Consequently, rectal surgery is generally considered to carry a lower pulmonary risk profile compared with more extensive upper abdominal procedures. The protective association observed in our model is therefore consistent with the expected gradient of pulmonary vulnerability across different abdominal surgical sites and supports the inclusion of surgical location as a relevant predictor in postoperative risk stratification.

Prolonged invasive mechanical ventilation is a well-established risk factor for postoperative pulmonary infection [30]. Prior studies have shown that endotracheal intubation lasting more than three days independently increases the likelihood of pulmonary infection [31]. In our study, ventilation duration > 6 h was incorporated as an early predictor to identify patients at risk shortly after surgery. During invasive mechanical ventilation, endotracheal intubation necessitates analgesia and sedation, which suppress glottic closure and cough reflexes, impairing airway clearance and facilitating bacterial colonization. In addition, patient–ventilator asynchrony or intolerance to intubation may provoke vomiting and aspiration, further elevating the risk of pulmonary infection [32,33].

Weilei Dai et al. [30] developed a prediction model for pulmonary infection in ICU patients; however, their study did not differentiate subgroups within the ICU population and included a relatively small sample size of only 398 cases. Similarly, Chao Lu et al. [34] constructed a model for patients undergoing surgery for primary liver cancer, enrolling 505 patients, but also faced limitations due to insufficient sample size. Comparable predictive models have been explored in patients after neurosurgery [35]. Overall, prediction models specifically targeting critically ill patients following abdominal surgery remain scarce.

Notably, the risk factors identified in our study did not include age or upper abdominal surgeries, which have been reported as important predictors in previous research [36]. Prior studies have shown that procedures closer to the diaphragm—such as thoracic and upper abdominal surgeries—can impair diaphragmatic excursion, reduce lung volumes, and substantially increase the risk of postoperative pulmonary complications [36]. However, this association was not observed in our critically ill cohort. A plausible explanation is that most patients in our study had significant pre-existing comorbidities, including hypertension and COPD, which may exert stronger and more direct effects on postoperative pulmonary infection than surgical location alone. The influence of age may also be partially mediated through these comorbidities in elderly patients, suggesting that underlying medical conditions represent more proximal determinants of infection risk. Furthermore, critically ill patients are affected by a complex interplay of perioperative physiological disturbances, and the relative contribution of the surgical site may be attenuated once postoperative organ dysfunction, inflammatory responses, and ventilatory requirements are accounted for. These findings imply that, in high-acuity ICU populations, postoperative physiological status and baseline comorbidities may overshadow the impact of anatomical surgical location on pulmonary infection risk.

Because this was a retrospective study, several limitations should be acknowledged. First, some postoperative variables included in the model (e.g., invasive mechanical ventilation > 6 h) may reflect early physiological responses rather than true predisposing factors, which limits causal interpretation, although these early postoperative changes remain clinically useful for risk stratification. Second, reliable information on several perioperative variables—such as aspiration risk, sedation and analgesia strategy, emergency status, and perioperative antibiotic exposure—was not consistently available and therefore could not be incorporated into the analysis. In addition, due to the limitations of the retrospective dataset, ventilator-associated pulmonary infections could not be distinguished from non–ventilator-associated events. This limitation restricts our ability to conduct more granular analyses of infection subtypes.

This study benefits from a large sample size and the application of rigorous statistical methods, including Lasso and logistic regression analyses. Nevertheless, as a single-center retrospective study, its generalizability may be limited. Future multicenter, prospective investigations are warranted to validate these findings and further refine the predictive model.

## 5. Conclusions

This study identified 8 independent predictors of pulmonary infection in critically ill patients after abdominal surgery: male sex (vs. female), COPD, atrial fibrillation, hypertension, chronic renal insufficiency, elevated preoperative total bilirubin, rectal surgery, and invasive mechanical ventilation > 6 h. Based on these variables, we developed and internally validated an early postoperative risk stratification model, which demonstrated good discriminatory performance (AUC 0.734) and favorable calibration. The model is intended as an early postoperative risk assessment tool rather than a causal framework, and may assist clinicians in identifying high-risk patients for intensified monitoring and preventive strategies. Future multicenter, prospective studies are warranted to externally validate and further refine the model to enhance its clinical applicability.

## Figures and Tables

**Table 1:** Comparison of prognostic data in patients.

Prognostic Indicator	Pulmonary Infection Group (*n* = 390)	Non-Pulmonary Infection Group (*n* = 4462)	Statistical Value	*p*
In-hospital mortality [*n* (%)]	58 (15.1)	129 (2.9)	χ^2^ = 138.927	<0.001 *
Length of stay in ICU [days, M (QL, QU)]	2 (2, 5)	2 (2, 3)	Z = −9.791	<0.001 *
Total length of stay [days, M (QL, QU)]	26 (17, 40)	18 (14, 24)	Z = −12.514	<0.001 *
Total cost [thousand yuan, M (QL, QU)]	198 (114, 328)	114 (81, 165)	Z = −12.646	<0.001 *

*: *p* < 0.05; ICU: intensive care unit.

**Table 2:** Comparison of data between the two groups.

Factors	Pulmonary Infection Group (*n* = 390)	Non-Pulmonary Infection Group (*n* = 4462)	Statistical Value	*p*
Gender			χ^2^ = 18.950	<0.001 *
Male (*n*)	233	2153		
Female (*n*)	157	2309		
Age (years, $\bar{x}$ ± *s*)	67.29 ± 13.57	63.32 ± 14.07	t = 5.354	<0.001 *
BMI (kg/m^2^, $\bar{x}$ ± *s*)	23.55 ± 4.18	23.77 ± 3.91	t = −1.076	0.289
**Comorbidities and past medical history**				
COPD [*n* (%)]	97 (24.9)	255 (5.7)	χ^2^ = 7.260	0.007 *
Asthma [*n* (%)]	10 (2.6)	69 (1.5)	χ^2^ = 2.319	0.128
Atrial fibrillation [*n* (%)]	34 (8.7)	177 (4.0)	χ^2^ = 19.463	<0.001 *
Coronary heart disease [*n* (%)]	43 (11.0)	539 (12.1)	χ^2^ = 0.378	0.539
Diabetes mellitus [*n* (%)]	97 (24.9)	943 (21.1)	χ^2^ = 2.975	0.085 *
Hypertension [*n* (%)]	232 (59.5)	2003 (44.9)	χ^2^ = 30.758	<0.001 *
Cerebral infarction [*n* (%)]	44 (11.3)	333 (7.5)	χ^2^ = 7.299	0.007 *
Chronic renal insufficiency [*n* (%)]	13 (3.3)	75 (1.7)	χ^2^ = 5.500	0.019 *
HBV infection [*n* (%)]	35 (9.0)	399 (8.9)	χ^2^ = 0.000	0.983
Fatty liver disease [*n* (%)]	19 (4.9)	192 (4.3)	χ^2^ = 0.279	0.597
Chemotherapy [*n* (%)]	15 (3.8)	142 (3.2)	χ^2^ = 0.505	0.477
Smoking status [*n* (%)]	112 (28.7)	1071 (24)	χ^2^ = 4.325	0.038 *
**laboratory examination**				
Preoperative leukocyte [×10^9^/L, M(Q_1_, Q_3_)]	6.0 (4.7, 8.1)	5.8 (4.6, 7.3)	Z = −3.428	0.001 *
Preoperative hemoglobin [g/L, M(Q_1_, Q_3_)]	118 (98, 137)	123 (108, 137)	Z = −3.836	<0.001 *
Preoperative platelet [×10^9^/L, M(Q_1_, Q_3_)]	191 (129, 254)	205 (155, 266)	Z = −2.727	0.006 *
Preoperative albumin [g/L, M(Q_1_, Q_3_)]	36.9 (33.6, 39.7)	38.2 (35.0, 41.3)	Z = −5.674	<0.001 *
Preoperative creatinine [umol/L, M(Q1, Q3)]	69 (53, 84)	67 (55, 81)	Z = −0.148	0.882
Preoperative total bilirubin [umol/L, M(Q1, Q3)]	12.5 (8.7, 24.1)	12.2 (8.7, 18.0)	Z = −1.745	0.081 *
Postoperative CK-MB [ng/mL, M(Q1, Q3)]	1.5 (1.0, 2.8)	1.3 (0.8, 2.2)	Z = −5.231	<0.001 *
Postoperative BNP [pg/mL, M(Q1, Q3)]	117 (57, 223)	80 (40, 149)	Z = −8.011	<0.001 *
Postoperative PT [s, M(Q1, Q3)]	13.2 (12.1, 14.2)	13.1 (12.2, 14.2)	Z = −1.128	0.259
Postoperative APTT [s, M(Q1, Q3)]	31.9 (28.9, 34.7)	30.5 (28.1, 33.6)	Z = −5.375	<0.001 *
**Surgery and anesthesia**				
Pancreatic surgery [*n* (%)]	60 (15.4)	467 (10.5)	χ^2^ = 8.961	0.003 *
Stomach surgery [*n* (%)]	24 (6.2)	233 (5.2)	χ^2^ = 0.621	0.431
Biliary surgery [*n* (%)]	10 (2.6)	104 (2.3)	χ^2^ = 0.085	0.771
Liver surgery [*n* (%)]	60 (15.4)	753 (16.9)	χ^2^ = 0.572	0.450
Splenic surgery [*n* (%)]	9 (2.3)	74 (1.6)	χ^2^ = 0.899	0.353
Jejunum surgery [*n* (%)]	5 (1.3)	53 (1.2)	χ^2^ = 0.000	1.000
Ileum surgery [*n* (%)]	3 (0.8)	44 (1.0)	χ^2^ = 0.022	0.881
Colon surgery [*n* (%)]	67 (17.2)	650 (14.6)	χ^2^ = 1.943	0.163
Rectal surgery [*n* (%)]	9 (2.3)	285 (6.4)	χ^2^ = 10.486	0.001 *
Kidney surgery [*n* (%)]	20 (5.1)	354 (7.9)	χ^2^ = 3.968	0.046 *
Ureteral surgery [*n* (%)]	10 (2.6)	138 (3.1)	χ^2^ = 0.339	0.560
Bladder surgery [*n* (%)]	14 (3.6)	199 (4.5)	χ^2^ = 0.647	0.421
Uterine surgery [*n* (%)]	10 (2.6)	268 (6.0)	χ^2^ = 7.868	0.005 *
Ovarian surgery [*n* (%)]	8 (2.1)	326 (7.3)	χ^2^ = 15.451	<0.001 *
Laparoscopic surgery [*n* (%)]	65 (16.7)	958 (21.5)	χ^2^ = 4.704	0.030 *
Night surgery [*n* (%)]	31 (7.9)	179 (4.0)	χ^2^ = 13.426	<0.001 *
Intraperitoneal chemotherapy [*n* (%)]	53 (13.6)	750 (16.8)	χ^2^ = 2.691	0.101
Intraoperative thermoperfusion therapy [*n* (%)]	5 (1.3)	151 (3.4)	χ^2^ = 5.093	0.024 *
Microwave ablation [*n* (%)]	2 (0.5)	33 (0.7)	χ^2^ = 0.038	0.845
Operation duration [min, M(Q_1_, Q_3_)]	284 (184, 402)	265 (192, 365)	Z = −0.936	0.349
Total intraoperative intake [ml, M(Q1, Q3)]	4000 (2600, 5700)	3600 (2600, 5050)	Z = −0.824	0.410
Total intraoperative output [ml, M(Q1, Q3)]	1100 (520, 2250)	1100 (600, 2000)	Z = −2.204	0.028 *
Intraoperative blood loss [ml, M(Q_1_, Q_3_)]	300 (70, 800)	300 (100, 850)	Z = −2.437	0.015 *
Intraoperative transfusion [*n* (%)]	173 (44.4)	1646 (36.9)	χ^2^ = 8.539	0.003 *
Intraoperative norepinephrine infusion [*n* (%)]	55 (14.1)	451 (10.1)	χ^2^ = 6.128	0.013 *
Nerve block [*n* (%)]	1 (0.3)	39 (0.9)	χ^2^ = 1.003	0.317
Analgesic pump [*n* (%)]	103 (26.4)	1281 (28.7)	χ^2^ = 0.930	0.3935
Nasogastric tube [*n* (%)]	310 (79.5)	3217 (72.1)	χ^2^ = 13.137	<0.001 *
ASA grade ≥ III [*n* (%)]	216 (55.4)	1697 (38.0)	χ^2^ = 45.219	<0.001 *
Invasive mechanical ventilation duration > 6 h [*n* (%)]	171 (43.8)	1220 (27.3)	χ^2^ = 57.466	<0.001 *

*: *p* < 0.05, BMI: body mass index, COPD: chronic obstructive pulmonary disease, HBV: Hepatitis B Virus, CK-MB: creatine kinase MB, BNP: B-type natriuretic peptide, PT: prothrombin time, APTT: activated partial thromboplastin time, ASA: American Society of Anesthesiologists.

**Table 3:** Multivariate Logistic regression analysis.

Variables	β	Std. Error	Wald χ^2^	OR	OR 95%CI	*p*
Male sex (vs. female)	0.411	0.166	6.170	1.509	1.091–2.087	0.013 *
COPD	1.421	0.187	58.007	4.139	2.872–5.966	<0.001 *
Atrial fibrillation	0.841	0.270	9.699	2.320	1.366–3.939	0.002 *
Hypertension	0.625	0.156	16.018	1.869	1.376–2.539	<0.001 *
Chronic renal insufficiency	0.881	0.381	5.338	2.412	1.143–5.091	0.021 *
Preoperative hemoglobin	−0.007	0.004	2.460	0.993	0.985–1.002	0.117
Preoperative platelet	−0.001	0.001	1.968	0.999	0.997–1.000	0.161
Preoperative albumin	−0.015	0.018	0.649	.985	0.951–1.021	0.421
Preoperative total bilirubin	0.003	0.001	9.508	1.003	1.001–1.004	0.002 *
Postoperative BNP	0.000	0.000	1.060	1.000	1.000–1.001	0.303
Postoperative APTT	0.012	0.007	3.022	1.012	0.998–1.026	0.082
Rectal surgery	−1.039	0.435	5.705	0.354	0.151–0.830	0.017 *
Intraoperative transfusion	0.228	0.158	2.085	1.256	0.922–1.711	0.149
Nasogastric tube	0.338	0.214	2.495	1.401	0.922–2.130	0.114
ASA ≥ III	0.198	0.164	1.457	1.218	0.884–1.679	0.227
Invasive mechanical ventilation duration > 6 h	0.791	0.155	26.017	2.206	1.628–2.990	<0.001 *

*: *p* < 0.05, COPD: chronic obstructive pulmonary disease, BNP: B-type natriuretic peptide, APTT: activated partial thromboplastin time, ASA: American Society of Anesthesiologists.

## Data Availability

The data presented in this study are available on request from the corresponding author due to privacy and ethical restrictions, as the clinical dataset contains potentially identifiable patient information.

## Abbreviations

ICU: intensive care unit; APACHE II: acute physiology and chronic health evaluation II; OR: odds ratio; CI: confidence interval; ROC: receiver operating characteristic; BMI: body mass index; COPD: chronic obstructive pulmonary disease; HBV: hepatitis B virus; CKMB: creatine kinase MB; BNP: B-type natriuretic peptide; PT: prothrombin time; APTT: activated partial thromboplastin time; ASA: American Society of Anesthesiologists; SD: standard deviation; IQR: interquartile range; DCA: decision curve analyses.
